# Antioxidant and antimicrobial activities of tarragon and *Zataria multiflora* Boiss essential oils and their applications as active agents to improve the shelf life of freshly cut potato strips

**DOI:** 10.1002/fsn3.3995

**Published:** 2024-01-30

**Authors:** Niyoosha Khajeh, Abdorreza Mohammadi Nafchi, Leila Nouri

**Affiliations:** ^1^ Food Science and Technology Department, Damghan Branch Islamic Azad University Damghan Iran; ^2^ Food Technology Division, School of Industrial Technology Universiti Sains Malaysia Penang Malaysia; ^3^ Green Biopolymer, Coatings & Packaging Cluster, School of Industrial Technology Universiti Sains Malaysia Penang Malaysia

**Keywords:** active coating, antioxidant activity, essential oil, microbiology, potato

## Abstract

This study investigated the possibility of using active coatings based on Zedo gum containing essential oils of *Zataria multiflora* Boiss (ZE) and tarragon (TE) to increase the shelf life and maintain the quality of freshly cut potato strips. The chemical compositions of ZE and TE were initially identified, and their antioxidant and antimicrobial activities were investigated. ZE consisted mainly of carvacrol (26.26%), p‐cymene (21.50%), thymol (18.05%), and linalool (11.31%), and those of TE comprised p‐allylanisole (81.92%), β‐Ocimene E (8.06%), and β‐Ocimene Z (5.35%). Afterwards, a Zedo gum active coating (1% v/w) containing 1% (v/v) essential oil was prepared, and the produced coating solutions were used to soak the potato strips for 5 min. The coated potatoes were kept fresh in a refrigerator for 9 days, and their quality characteristics were examined every 3 days. The results show that the weight loss, browning index, total microbial count, and mold and yeast counts in the strips increased during 9 days of cold storage, and hardness decreased (*p* < .05). However, the coatings of strips, especially those containing essential oils, reduced the intensity of changes in moisture, color, and hardness, increased microbial stability, and maintained the sensory acceptance of strips compared with the uncoated sample (control). Finally, this study demonstrated that the quality and shelf life of fresh potato strips can be improved by using active coatings based on Zedo gum containing ZE and TE (especially ZE).

## Graphical abstract

The chemical compositions of ZE and TE were initially identified, and their antioxidant and antimicrobial activities were investigated. The coatings of strips, especially those containing essential oils, reduced the intensity of changes in moisture, color, and hardness, increased microbial stability, and maintained the sensory acceptance of strips compared with the uncoated sample.

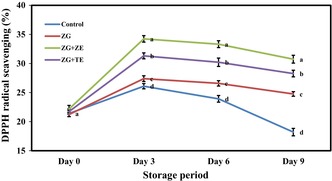

## 1 INTRODUCTION

The consumption of fruits and vegetables, considered rich sources of fiber, has increased rapidly due to their contents of various essential micronutrient compounds, such as vitamins (Wijekoon et al., 2023). Fruits and vegetables contain high amounts of moisture and are rich food products, and thus, they are highly prone to chemical and biological spoilage; breathing can also damage the quality of these crops (Esmaeili et al., 2021). Enzymatic browning is another problem in some crops, such as fresh‐cut potatoes and apples, adversely affecting the nutritional value and sensory quality of these products. Enzymatic browning results from the oxidation of phenolic compounds by polyphenol oxidase (Qiao et al., 2021). Thus far, various methods have been proposed to extend the shelf life of these perishable foods, with the use of edible coatings considered one of the most efficient and interesting (Oladzadabbasabadi, Mohammadi Nafchi, Ghasemlou, et al., 2022).

Edible coatings are thin polymer layers that adhere to food surfaces. These can be prepared from various biopolymers, such as proteins, polysaccharides, or their combination (Oladzadabbasabadi, Mohammadi Nafchi, Ariffin, et al., 2022; Shabahang et al., 2022). These coatings are prepared as viscose solutions and applied to products through spraying or dipping (Díaz‐Montes & Castro‐Muñoz, 2021). Applying edible coatings to food surfaces can maintain the moisture of food products and reduce the growth of microorganisms and destructive reactions. On the other hand, these coatings can also carry active agents, such as antimicrobials and antioxidant agents, and release them onto food surfaces (Esfahani et al., 2022; Liu et al., 2022; Samani et al., 2023). Zedo gum is an anionic arabinose–galactomannan that contains 2.45% protein and 0.85% fat and has a dry weight consisting mainly of polysaccharides (92.36%). This gum, also known as Shirazi or Persian gum, is obtained from mountain almond trees (Khalesi et al., 2016). Zedo gum contains different chemical compounds that render it various colors (white, yellow, and red) (Razmjoo et al., 2021). Zedo gum comprises water‐insoluble (70%–75%) and water‐soluble (25%–30%) parts (Hadian et al., 2016). Its structure is similar to that of Arabic gum, and it contains high amounts of galactose and arabinose and small amounts of rhamnose and galacturonic acid (Fadavi et al., 2014). This gum is less expensive than other gums, such as xanthan, Arabic gum, and locust bean gum, and is biodegradable and nontoxic (Razmjoo et al., 2021).

Herbal essential oils and extracts are considered natural preservative sources that contain various amounts of phytochemical compounds (Bagher Abiri et al., 2023; Nikmanesh et al., 2023). Essential oils can improve the functional properties of edible coatings and films and often exhibit remarkable antifungal, antioxidant, and antibacterial activities (Babapour et al., 2021; Shahidi & Hossain, 2022). Tarragon is a medicinal plant from the Asteraceae family (Azizkhani et al., 2021). This plant contains essential oils (0.15%–3.10%), whose amount and composition depend on the material origin. Estragole is the most abundant bioactive compound in tarragon essential oil (TE), accounting for more than 74% of its compounds (Socaciu et al., 2020). The considerable antioxidant and antimicrobial activities of TE in edible coatings and films have been reported (Socaciu et al., 2021). *Zataria multiflora* Boiss, also known as Shirazi thyme, is an aromatic plant that belongs to the Lamiaceae family. This plant contains high amounts of major bioactive compounds, including thymol, carvacrol, γ‐terpinene, and p‐cymene (Pourhosseini et al., 2020). The antioxidant, antifungal, and antibacterial activities of thyme essential oil have been investigated and confirmed by previous researchers (Chang et al., 2021; Mousavian et al., 2021); this essential oil has been successfully applied in the development of active films and coatings for increased shelf life and quality of food products (Chang et al., 2022; Hematizad et al., 2021). To the best of our knowledge, active coatings based on Zedo gum and these essential oils on the characterization of fresh‐cut potato strips have not been investigated yet. Therefore, this research focused on investigating the effects of active and edible coatings based on Zedo gum containing *Zataria multiflora* Boiss essential oil (ZE) and TE on enzymatic browning, antioxidant activity, physical and sensory properties, and microbial load of fresh‐cut potato strips during the cold storage period.

## 2 MATERIALS AND METHODS

### 2.1 Materials

The Zedo gum was purchased from Giahine Company (Esfahan, Iran). The bacterial strains were prepared at the Iran Industrial Microorganism Collection Center. The culture medium, antibiotics, and chemicals used to conduct experiments were bought from Merck Company (Germany).

### 2.2 Preparation of essential oils and analysis of their chemical composition

*Zataria multiflora* Boiss and tarragon plants were collected from the lands of Damghan Agricultural University (Iran), and their leaves were separated and dried for 7 days in the dark. ZE and TE were extracted through distillation using water in a Clevenger system at a plant powder‐to‐distilled water ratio of 1:7. The extraction of essential oils lasted for 3 h (Zaïri et al., 2020).

The separation and identification of chemical constituents of essential oils were performed on a Hewlett‐Packard 6890‐5972GC‐MS system equipped with an FID detector and an HP‐5MS capillary column (30 m length × 0.25 mm diameter × 0.25 μm thickness of the inner layer). Pure helium gas with a flow rate of 1.5 mL/min was used as the carrier gas. The injection, ion source, and interface temperatures were all set at 250°C. The temperature program used for the column was 60°C–246°C with a rate of 3°C/min, and the end temperature was set at 250°C for 10 min. Ionization of the sample components was performed at 70 eV. The split ratio was 90:1 (Oualdi et al., 2022).

### 2.3 Antioxidant activity of essential oils

The antioxidant activity of different concentrations of TE and ZE was evaluated using the 2,2‐diphenyl‐1‐picrylhydrazyl (DPPH) radical‐scavenging method and the β‐carotene bleaching assay. DPPH radical scavenging by the essential oils was measured using the method described by Dalli et al. (2021) with some modifications. First, methanolic solutions of TE and ZE were prepared at concentrations of 1–8 mg/mL. Each essential oil (50 μL) was then mixed with 0.004% DPPH methanolic solution (5 mL), and the mixture was stirred well. Afterwards, the prepared mixtures were incubated at room temperature in the dark for 30 min. Finally, the absorbance of the mixtures was recorded at 517 nm. Ascorbic acid (vitamin C) was used as the standard antioxidant in this assay, and methanol was used as a blank. The percentage of DPPH radicals scavenged by the essential oils was determined using the following equation:

$$\text{DPPH}\;\left(\%\right)=\frac{\text{Blank absorbance}-\text{Sample absorbance}}{\text{Blank absorbance}}\times 100$$

To measure the antioxidant activity of essential oils via the β‐carotene bleaching assay, we initially dissolved 𝛽‐carotene (2 mg) in chloroform (10 mL) and added linoleic acid (20 mg) and Tween‐80 as an emulsifier (200 mg) to the solution. Chloroform was removed from the solution at 40°C, and distilled water (100 mL) was added with vigorous stirring. Afterwards, TE and ZE were dissolved in methanol and transferred to test tubes at different concentrations (0.2 mL of each concentration) along with the resulting emulsion (1.80 mL). The prepared tubes were incubated for 2 h at 50°C in a water bath with continuous shaking, and the first absorbance of the solutions was recorded immediately (t_0_) at 470 nm. After 2 h, the absorbance was recorded again (t_2_). Ascorbic acid (vitamin C) was used as the standard antioxidant in this assay. The percentage of β‐carotene bleaching inhibited by the essential oils was calculated using the following equation (Oualdi et al., 2022):

$$\beta -\text{carotene bleaching}\;\left(\%\right)=100–\left[\left(\frac{t0-t2}{t0}\right)\times 100\right]$$

### 2.4 Antibacterial activity of essential oils

The minimum inhibitory concentration (MIC) and minimum bactericidal concentration (MBC) of the essential oils on bacterial strains of *Staphylococcus aureus* (PTCC1189), *Escherichia coli* (PTCC1108), *Listeria monocytogenes* (PTCC1163), and *Shigella dysenteriae* (PTCC1188) in 96‐well plates were determined using the broth microdilution method. First, Muller Hinton Broth medium (100 μL) was poured into the microplates, and essential oil (100 μL) was added to the first well. Starting from the first well, the mixture was diluted until the ninth well. Antibiotics suitable for the sensitivity of the tested bacteria (100 μL), including ciprofloxacin, tetracycline, and gentamicin, were added in another row. Finally, a diluted microbial suspension equal to 0.5 McFarland (100 μL) was added to all wells. Different concentrations of essential oils were prepared through the dilution of the original solution, which was prepared using 1000 μg/mL dimethyl sulfoxide, and used to obtain different concentrations of essential oils (16.25, 32.5, 65, 125, 250, 500, and 1000 μg/mL). After 24 h of incubation at 37°C, the bottom of the plate was observed under a light in the mirror. Turbidity indicates the growth or non‐growth of bacteria. Based on the definition, the MIC was determined in the last well, which showed no turbidity. For the MBC test, all the wells without turbidity were cultured separately on Muller Hinton Agar medium, and after 24 h, the concentration at which bacterial growth was not observed was reported as the MBC (Donsì et al., 2011).

The antibacterial activity of essential oils was also investigated by the agar disk diffusion method. In this way, after preparing the bacterial suspensions (8 log CFU/ml) and spreading them uniformly on the Trypticase Soy Agar (TSA) culture media, discs with a diameter of 6 mm immersed in essential oils (5 μL) were placed on the surface of the culture media. The plates were kept in an incubator at 37°C for 24 h, and then the diameter of the inhibition zone formed on the plates was measured (Sarengaowa et al., 2022).

### 2.5 Preparation of active coatings and coated freshly cut potato strips

To prepare the coating solutions, we first added 1% w/v Zedo gum to deionized water at normal temperature and stirred the solution using a high‐speed stirrer for 30 min to ensure uniformity. Afterwards, the resulting solution was kept at room temperature for 24 h for the maximum absorption of water by the Zedo gum. The solution was then centrifuged (Sigma 1–14, Germany) for 15 min at 33000 × g. Finally, the supernatant was separated and used as a coating. ZE and TE (1% v/v) were added separately to the 1% Zedo gum solution and emulsified using a homogenizer (IKA SS42, Germany) for 5 min at 12000 rpm (Barzegar et al., 2019).

The potatoes were washed, peeled, and cut into strips (1 × 1 × 8 cm^3^) using a cutter. The potato strips were then washed and soaked in distilled water to remove starch. Then, enzyme removal was performed at 85°C for 6 min. The strips were immersed in active coating solutions for 5 min, and the control sample was in distilled water. The strips and coating solutions had a ratio of 1 g to 4 mL. After this point, the samples were removed from the solutions and dried at room temperature. The strips were packed in zipped polyethylene bags and stored for 9 days at refrigerator temperature (4 ± 1°C) and a relative humidity of 95%, with tests conducted on them every 3 days.

### 2.6 Analysis of weight loss

To measure the weight loss of samples, we weighed the strips every 3 days and calculated the weight loss percentage through the differences between the initial and final weights (Ruelas‐Chacon et al., 2017).

### 2.7 Analysis of texture

The hardness of the potato strips was measured using a Brookfield Texture Analyzer (England). The samples were compressed using a cylindrical probe (50 mm diameter) at a speed of 1 mm/s until they reached 55% of their initial height (Rizzo et al., 2018).

### 2.8 Analysis of browning index (BI)

The BI of the potato strips was determined using a ColorFlex Hunterlab system (America). The color indices of L*, a*, and b* were determined, and the BI was calculated using the following equation (Velickova et al., 2014):

$$\mathrm{BI}=100/0.17\;\left(\frac{a*+1.75\;L*}{5.645L*+a*-3.012b*}\right)-0.31$$

### 2.9 Analysis of antioxidant activity

An antioxidant activity test was performed on methanolic extracts of potato strips. To prepare the extract samples, we mixed the homogenized potato (5 g) with 80% methanol solvent (50 mL) and stirred the mixture on a shaker (Heidolph, Germany) at room temperature for 60 min and 1000 rpm. The resulting extracts were finally filtered using a Whatman filter paper (No. 1).

The antioxidant activity of the potato strips was determined using the DPPH radical‐scavenging method. The extract (0.1 mL) was mixed with 0.24 g/L DPPH methanolic solution (3.9 mL) and stored in the dark for 30 min at room temperature. Afterwards, the absorbance of the solution was recorded at a wavelength of 515 nm using an ultraviolet (UV)‐visible spectrophotometer (UV–Visible s2150, Unico, America). The DPPH radical‐scavenging activity of the samples was calculated using the following equation:

$$\text{DPPH}\;\left(\%\right)=\frac{\mathrm{Ac}-\mathrm{As}}{\mathrm{Ac}}\times 100$$

where Ac and As refer to the absorbance of the control sample at the beginning of the test and the absorbance of the tested sample after 30 min, respectively (Licciardello et al., 2018).

### 2.10 Microbial analysis

For the microbial tests, we first prepared the desired dilutions from each sample. The chopped potato (10 g) was mixed with peptone water buffer (0.1%) at a ratio of 1:10 (w/v) for 3 min. Other dilutions were prepared in the same manner. Plate count agar and potato dextrose agar medium were used for the total bacterial count and mold and yeast count, respectively. Incubation of the cultured plates for the total microbial count was conducted at 30°C for 48 h and at 25°C for 5 days for the mold and yeast counts. The microbial load results were reported as the logarithm of colony number per gram of sample (log CFU/g) and the counting of molds and yeasts as CFU/g (Shehata et al., 2020).

### 2.11 Sensory acceptance evaluation

The sensory acceptance evaluation of the potato strips was performed using the 5‐point hedonic scale (1: very bad to 5: very good). After sample coding, ten panelists, while considering the appearance, color, texture, and odor of the potato strips, rated their overall sensory acceptance.

### 2.12 Statistical analysis

The tests were examined with 3 replications, and their statistical analysis was performed using the SPSS software (version 28.0) and ANOVA (one‐way analysis of variance). The sample differences were studied at *p* < .05 using Duncan's multiple range test.

## 3 RESULTS AND DISCUSSION

### 3.1 Chemical constituents of essential oils

Tables 1 and 2 present the chemical constituents of ZE and TE, respectively. Twenty‐three phytochemicals were identified in ZE, thymol (26.00%), the highest amounts of which were related to thymol (26.26%), p‐cymene (21.50%), carvacrol (18.05%), linalool (11.31%), β‐caryophyllene (4.60%), and α‐pinene (4.12%). Similarly, Rahimi et al. (2019) isolated thymol (34.44%), carvacrol (33.45%), and p‐cymene (15.62%) as major chemical constituents of ZE. In another study, thymol was the major component of ZE (Radünz et al., 2021). Twenty‐one phytochemical compounds were identified in TE, the most important of which were estragole (81.92%), β‐ocimene E (8.06%), and β‐ocimene Z (5.35%). These results were consistent with those obtained by Azizkhani et al. (2021), who also reported estragole as the major component of TE. The comparison of the major components of TE and ZE in the present study with those of previous research indicated the varying amounts of active compounds in different samples. This difference was probably due to the variations in the variety, cultivation conditions, weather conditions, harvest time, and storage conditions of the samples (Chaleshtori et al., 2013).

**TABLE 1:** Chemical constituents of ZE analyzed by gas chromatography–mass spectrometry (GC–MS).

Component	Retention time	Amount (%)
α‐Thujene	933	0.09
α‐Pinene	940	4.12
Camphene	961	0.18
β‐Pinene	981	0.55
β‐Myrcene	990	1.08
3‐Octanol	996	0.30
p‐Cymene	1031	21.5
Limonene	1033	1.22
1,8‐Cineole	1035	0.87
y‐Terpinolene	1066	0.16
Linalool	1100	11.31
Borneol	1153	0.25
4‐Terpineol	1180	0.41
α‐Terpineol	1192	0.74
Thymol methyl ether	1233	1.02
Carvacrol methyl ether	1254	3.95
Carvacrol	1303	18.05
Thymol	1295	26.26
β‐Caryophyllene	1431	4.60
Aromadendrene	1441	1.32
Alpha‐humulene	1473	0.36
Ledene	1502	0.63
Caryophyllene oxide	1591	1.42

**TABLE 2:** Chemical constituents of TE analyzed by GC–MS.

Component	Retention time	Amount (%)
α‐Pinene	937	0.35
Sabinene	969	0.02
β‐Pinene	978	0.09
β‐Myrcene	989	0.02
α‐Phellandrene	1008	0.06
Limonene	1030	1.13
β‐Ocimene Z	1035	5.35
β‐Ocimene E	1049	8.06
α‐Terpinolene	1075	0.04
Linalool	1102	0.02
Allo‐Ocimene	1129	0.11
Estragole	1222	81.92
Carvone	1250	0.15
α‐Terpineol	1258	0.04
Borneol	1268	0.02
β‐Bisabolene	1271	0.01
I‐Bonyl acetate	1292	0.08
Caryophyllene oxide	1324	0.01
Methyleugenol	1425	0.41
Spathulenol	1573	0.02
Carvacrol	1724	0.01

### 3.2 Antioxidant activity of essential oils in vitro

**FIGURE 1:**
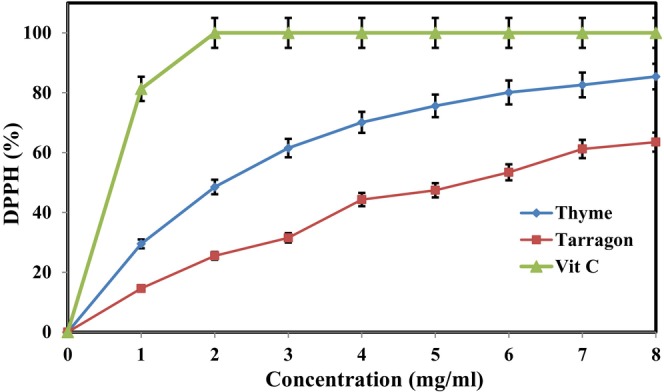
Comparison of changes in DPPH radical scavenging (%) of TE and ZE in different concentrations.

**FIGURE 2:**
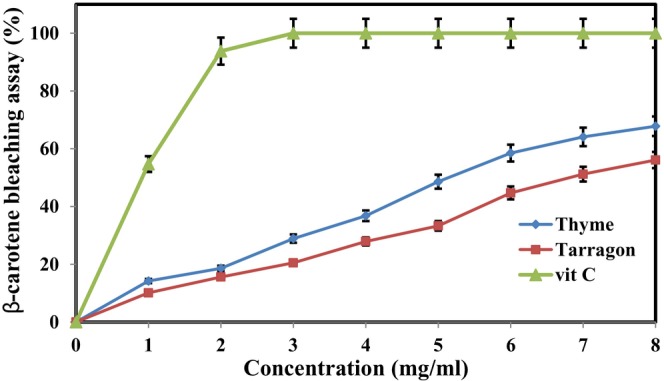
Comparison of changes in the β‐carotene bleaching assay (%) of TE and ZE in different concentrations.

In this research, the antioxidant activities of different concentrations of TE and ZE were evaluated using the DPPH radical scavenging method and the β‐carotene bleaching assay and compared with those of ascorbic acid (vitamin C) as the standard antioxidant. DPPH is a free and unstable radical, and its maximum absorbance can be observed at the wavelength of 517 nm. Any compound that can donate hydrogen can modify DPPH from its radical state and convert it into a stable form, the state of which is determined through the changes in the color of the DPPH solution and its absorbance (Hadidi et al., 2020). The β‐carotene bleaching assay is based on the decolorization of β‐carotene, whose mechanism involves the reaction of β‐carotene with the free radicals produced by the formation of hydroperoxide from linoleic acid. The rate of β‐carotene decolorization decreases in the presence of antioxidants (Yeddes et al., 2019). Figures 1 and 2 show the changes in DPPH free‐radical scavenging and β‐carotene bleaching activities of TE and ZE at different concentrations, respectively. ZE demonstrated higher antioxidant activity than TE. A direct and positive relationship was observed between the concentration of essential oils and their antioxidant activities. Thus, with the increase in the concentration of both essential oils, the percentages of DPPH free radical scavenging and β‐carotene bleaching increased. The percentages of DPPH free‐radical scavenging in the 8 mg/mL concentration of ZE and TE reached 85.4% and 63.5%, respectively, and those of β‐carotene bleaching of both essential oils were 67.8% and 56.1%. The antioxidant activities of these essential oils showed considerable differences with those of vitamin C at all concentrations. The antioxidant activity of vitamin C reached 100% at a concentration of 2 mg/mL.

Similarly, Alsaraf et al. (2020) and Azizkhani et al. (2021) considered the antioxidant activities of ZE and TE to be concentration‐dependent. They reported an increase in their DPPH radical scavenging as a result of increased concentration. Ed‐Dra et al. (2021) also showed the high antioxidant activity of thyme essential oil in the DPPH radical‐scavenging method, β‐carotene bleaching assay, and iron‐reducing method. Raeisi et al. (2019) studied the antioxidant activity of ZE and reported the β‐carotene bleaching capability of this essential oil at 66.46%, which is consistent with the results reported for ZE (8 mg/mL) in the present study.

### 3.3 Antibacterial activity of essential oils

Table 3 provides the MIC and MBC of TE and ZE against the studied bacteria. The lowest MIC (31.2 μg/mL) and MBC (62.5 μg/mL) of ZE were related to *S. aureus* bacteria, and the highest (125 and 250 μg/mL, respectively) were obtained against *Sh. dysenteriae* and *E. coli*. The lowest and highest MIC (62.5 and 250 μg/mL, respectively) and MBC (125 and 500 μg/mL) of TE were observed against *S. aureus* and *Sh. dysenteriae*, respectively. The MBC was constantly equal to or greater than the MIC. Raeisi et al. (2019) reported that the MIC of ZE against different bacteria ranged from 156.2 ppm to 625 ppm. They also considered the lowest MIC to be related to *S. aureus*. Examining the antibacterial activity of essential oils using the agar disk diffusion method (Table 3) also showed a higher antibacterial activity of essential oils against Gram‐positive bacteria compared to Gram‐negative bacteria. ZE indicated the highest activity compared with TE, so the values of the diameter inhibitory zone of ZE against the bacterial strains examined in its research were in the range of 17.80–29.41 mm, while its values for TE were in the range of 11.59–23.10 mm. In general, Gram‐positive bacteria are more sensitive to essential oils than Gram‐negative bacteria because, in the latter, a thick layer of peptidoglycan surrounds the outer membrane of cells, which indicates a protective effect (Guimarães et al., 2019). Azizkhani et al. (2021) also reported the substantial antibacterial activity of TE against *S. aureus*, *L. monocytogenes*, *E. coli*, and *Sh. dysenteriae* and observed the higher activity of this essential oil against Gram‐positive bacteria than Gram‐negative bacteria. In Gedikoğlu et al. (2019) study, the Gram‐positive *S. aureus* exhibited a higher antibacterial activity than the Gram‐negative *E. coli*. Another study reported the remarkable antibacterial activity of ZE against food pathogens (Hematizad et al., 2021). Essential oils are known for their hydrophobicity, which allows them to easily penetrate the lipids of the bacterial cell membrane, disrupt the cell structure, and increase permeability. An increase in membrane permeability leads to the release of intracellular components to the external environment, affects bacterial viability, and results in cell death (Burt, 2004; Hematian et al., 2023).

**TABLE 3:** MIC and MBC of TE and ZE in the studied bacteria.

Bacteria	ZE			TE		
	Inhibitory diameter zone (mm)	MIC (μg/ml)	MBC (μg/ml)	Inhibitory diameter zone (mm)	MIC (μg/ml)	MBC (μg/ml)
*S. aureus*	29.41 ± 0.32 A,a	31.2	62.5	23.10 ± 0.54 A,b	62.5	125
*Sh. dysenteriae*	17.80 ± 0.46 D,a	125	250	11.59 ± 0.18 D,b	250	500
*L. monocytogenes*	27.63 ± 0.25 B,a	62.5	125	19.76 ± 0.37 B,b	125	250
*E. coli*	18.95 ± 0.38 C,a	125	250	14.87 ± 0.31 C,b	125	500

Abbreviations: MIC, Minimum inhibitory concentration; MBC, minimum bactericidal concentration; TE, tarragon essential oil; ZE, *Zataria multiflora* essential oil.

*Note*: Different capital and small letters mean significant differences at the 5% level of probability among bacterial strains and essential oils, respectively.

### 3.4 Weight loss of potato strips

**FIGURE 3:**
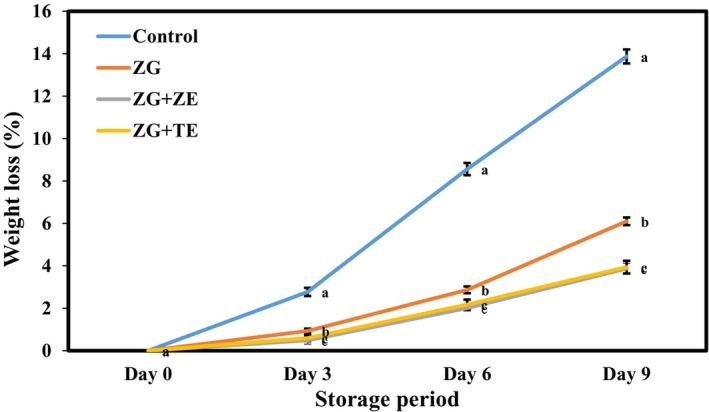
Changes in weight loss (%) of freshly cut potato strips during 9 days of cold storage. Different letters represent significant differences at the 5% level of probability among samples. ZG + TE, Zedo gum‐based coating containing tarragon essential oil; ZG + ZE, Zedo gum‐based coating containing *Zataria multiflora* essential oil; ZG, Zedo gum‐based coating.

Weight loss, which indicates the freshness of food products, is a noticeable quality parameter in agricultural product storage. Freshly cut products are more sensitive to weight loss than whole products because cutting vegetables and fruits increases their surface area‐to‐volume ratio and allows the rapid release of moisture (Yousuf & Srivastava, 2017). Figure 3 shows the changes in the weight loss values of fresh potato strips during a 9 days storage period in the refrigerator. Over time, the weight loss of samples significantly increased (*p* < .05), and the highest rate of increase was observed in the uncoated sample (control). The weight loss of this sample reached 2.77% on the third day of storage and 13.87% on the last day. In the sample coated with Zedo gum without essential oil, the weight loss was 0.93% on the third day, and its value increased to 6.10% on the last day. The lowest weight changes occurred in the samples coated with solutions containing TE and ZE, with values of 0.49% and 0.59%, respectively, at the beginning and 3.87% and 3.94% on the last day of storage. Edible coatings and films serve as barriers against moisture and oxygen (Basaglia et al., 2021). Thus, with the application of Zedo gum coating on freshly cut potato strips, the rate of moisture transfer decreased, and more moisture was retained in the product.

On the other hand, with the reduction of available oxygen, the respiration rate of potatoes and their weight loss decreased. Through the addition of TE and ZE to the coating solutions, given the hydrophobic properties of essential oils, the barrier activity of the coatings was improved, and the weight loss of samples was reduced via reduced moisture and oxygen passage. Other researchers also observed the increased weight loss of fruits and vegetables during the storage period (Babapour et al., 2022; Zhang et al., 2019). Moreover, the important effect of active coatings and films on the reduction of the weight loss of coated products compared with the uncoated samples was previously observed. The reduced weight loss of freshly cut potatoes caused by the use of an edible coating based on cactus polysaccharides was observed by Wu (2019). The significant effect of edible biopolymer coatings on reducing the percentage of weight loss of freshly cut potatoes was also reported in Bari and Giannouli (2023).

### 3.5 Hardness of potato strips

**FIGURE 4:**
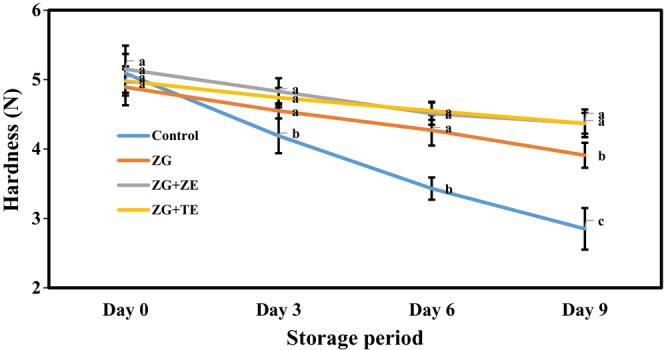
Changes in hardness (*N*) of freshly cut potato strips during 9 days of cold storage. Different letters represent significant differences at the 5% level of probability among samples. ZG + TE, Zedo gum‐based coating containing tarragon essential oil; ZG + ZE, Zedo gum‐based coating containing *Zataria multiflora* essential oil; ZG, Zedo gum‐based coating.

Hardness is another important and effective characteristic of the quality and shelf life of freshly cut vegetables and fruits (El‐Mogy et al., 2019). Figure 4 shows the changes in the hardness of different freshly cut potato strips during the storage period. During the initial storage, the active coating containing essential oils caused a slight and insignificant decrease in the hardness of the strip samples, whose hardness values were in the range of 4.89–5.15 N. Over time, the hardness of the potato strips decreased gradually. Still, their coating caused a significant decrease in the intensity of textural changes in the samples compared with the control (*p* < .05). Thus, on the last day of storage, the control sample had a hardness of 2.85 N, and the samples coated with Zedo gum without essential oil and gum solutions containing ZE and TE attained hardness values of 3.91, 4.37, and 4.37 N, respectively. The decrease in hardness over time was probably due to the breathing of potatoes and the release of tissue‐decomposing enzymes (Salehi & Sheikhzadeh, 2019). During the respiration of vegetables and fruits, pectin is decomposed, starch is hydrolyzed, and their texture becomes softer. Therefore, active coatings based on Zedo gum and essential oils reduce the intensity of respiration and prevent texture destruction of the potato strips, which reduces the intensity of texture softening. Ali et al. (2021) also observed that coating potato strips with edible coatings based on garden cress mucilage alone or in combination with ascorbic acid can considerably decrease the softening rate of the sample texture during the storage period compared with the uncoated sample. Sumonsiri et al. (2020) observed that the hardness of potatoes coated with a pectin solution containing sweet orange and lemon essential oils was significantly higher than that of the uncoated sample on the seventh day of cold storage.

### 3.6 BI of potato strips

**FIGURE 5:**
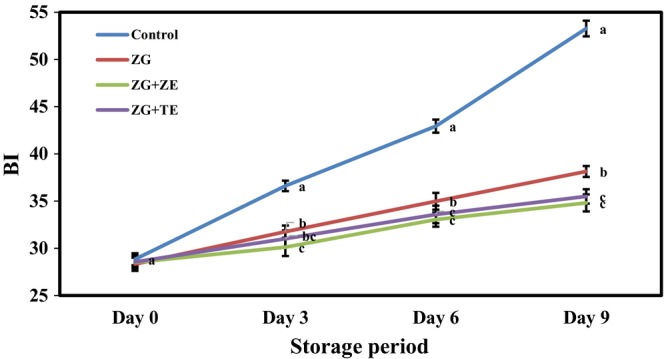
Changes in BI of freshly cut potato strips during 9 days of cold storage. Different letters represent significant differences at the 5% level of probability among samples. BI, Crowning index; ZG + TE, Zedo gum‐based coating containing tarragon essential oil; ZG + ZE, Zedo gum‐based coating containing *Zataria multiflora* essential oil; ZG, Zedo gum‐based coating.

Browning is a major problem in the food industry; it can affect the sensory characteristics of food products and decrease the economic value and quality of foods (Chen et al., 2005). Cutting and peeling of crops, such as potatoes, lead to cell tissue damage, and the release of the polyphenol oxidase enzyme, especially tyrosinase, from the cells leads to browning of the surface of food products (Sumonsiri et al., 2020). The enzymatic browning reaction is carried out in the presence of oxygen, and as a result, monophenols are oxidized to diphenols and eventually into O‐quinones. On the other hand, O‐quinones can be oxidized to other polyphenols (Jung & Choi, 2020). Figure 5 displays the changes in the BI of freshly cut potato strips during the 9 days cold storage period. On the first day of storage, the potato strips had BI values in the range of 28.33–28.84, which showed no statistically significant difference between them. Over time, the BI values of the strip samples increased significantly (*p* < .05).

However, coating the strips with active coatings based on Zedo gum, especially the solutions containing ZE and TE, led to a significant reduction in the rate of browning (*p* < .05). The reduction in the intensity of the browning of potatoes because of the use of edible coatings is related to the oxygen barrier of the coatings (Wu, 2019). Oxygen serves as an important and effective parameter of the intensity of the enzymatic browning of food products. Therefore, limiting the access to oxygen through the application of edible coatings can reduce the extent of browning. Furthermore, the addition of essential oils to coating solutions can improve the gas barrier capability of coatings and their effect on the browning of potatoes. In addition, given the antioxidant activity of Zedo gum, TE, and ZE, the use of active coatings based on these compounds can reduce the rate of oxidation of phenolic compounds and, therefore, also reduce the intensity of browning in the samples. Liu et al. (2019) also observed that the purslane extract, which is rich in antioxidant compounds, can successfully reduce the rate of browning in freshly cut potatoes during the storage period. Ali et al. (2021) achieved similar results. They reported the reduced BI of potato strips caused by the use of edible coatings based on garden cress, especially those containing the active antioxidant ascorbic acid. The effect of an alginate‐based edible coating enriched with thyme essential oil on reducing the BI of freshly cut potatoes was also observed in the research of Sarengaowa et al. (2022). Bari and Giannouli (2023) stated that mucilaginous coatings can act as protective layers on product surfaces and prevent color changes over time.

### 3.7 Antioxidant activity of potato strips

**FIGURE 6:**
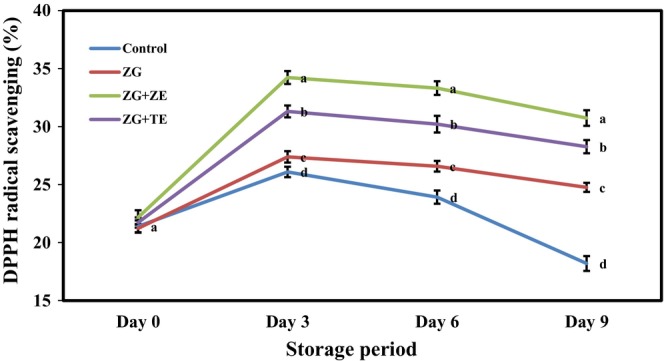
Changes in DPPH radical scavenging (%) of freshly cut potato strips during 9 days of cold storage. Different letters represent significant differences at the 5% level of probability among samples. ZG + TE, Zedo gum‐based coating containing tarragon essential oil; ZG + ZE, Zedo gum‐based coating containing *Zataria multiflora* essential oil; ZG, Zedo gum‐based coating.

Fruits and vegetables contain high amounts of phenolic compounds and natural antioxidants, which can reduce the risk of various diseases (Bhattacharya et al., 2010). Phenolic compounds, such as gallic acid, vanillic acid, p‐coumaric acid, caffeic acid, chlorogenic acid, and protocatechuic acid, have been found in potato tubers, which proves its antioxidant activity (Kafi et al., 2019). The tissue damage and stress caused by cutting and peeling can lead to the increased accumulation of phenolic compounds through the activation of the phenylpropanoid pathway (Liu et al., 2019). The investigation of the antioxidant activity of freshly cut potato strips (Figure 6) revealed that no statistically significant difference was initially observed between the uncoated sample and those coated with the Zedo gum solution. In addition, the samples had DPPH radical‐scavenging rates in the range of 21.33%–22.17%. During cold storage, phenolic compounds had an initially higher formation rate than oxidation rate. Thus, the amounts of these compounds in the samples initially increased with time. Then, the rate of production gradually decreased compared with that of oxidation, and the antioxidant activity of the strips decreased. The antioxidant activity trend of freshly cut potato strips observed in this research was consistent with that reported by Dovene et al. (2019). The use of active coatings can considerably reduce the rate of decrease in the antioxidant activity of potato strips over time compared with the control because the coatings act as a barrier on the product and reduce the access to oxygen and the rate of enzymatic oxidation of phenolic compounds, which preserve the bioactive compounds (Dong & Wang, 2018); therefore, the coatings reduced the rate of reduction in the antioxidant activity over time. In the final phase of storage, the control sample had the lowest antioxidant activity (18.20%), and the highest value was obtained for the sample coated with the Zedo gum solution containing ZE (30.74%). These results were close to the expected findings because ZE and TE initially exhibited significant antioxidant activities, but that of ZE was higher. In line with the results of the present study, maintenance and improvement of the antioxidant activity of different fruits and vegetables after the application of gum‐based coating solutions on has been reported by previously (Hashemi et al., 2017; Tahir et al., 2019).

### 3.8 Microbial load of potato strips

Microbial load is one of the important parameters in guaranteeing the safety of freshly cut fruits and vegetables. As a result of the breathing of a product and the softening of its tissue during the storage period, the sensitivity to the growth of microorganisms increases, which results in their increased numbers in the product (Silveira et al., 2017). Most crops have a pH close to neutral values and high moisture, and thus, they can provide suitable substrates for the growth of various microorganisms, such as molds and bacteria (Alam et al., 2014). Table 4 shows the total microbial count and the mold and yeast counts of freshly cut potato strips during the cold storage period. The highest microbial load and mold and yeast counts were first observed in the control sample (2.15 log CFU/g and 2.33 CFU/g, respectively) and the sample coated with Zedo gum solution without essential oils (2.13 log CFU/g and 1.33 CFU/g). The use of active coatings containing ZE and TE led to a significant decrease in the total microbial load of the strips (*p* < .05), with the numbers of microorganisms in these samples reaching 1.74–1.75 log CFU/g. At this point, the samples coated with solutions containing essential oils did not contain molds and yeasts. Storage at refrigerator temperature also resulted in a remarkable effect on the microbial load and the number of molds and yeasts in the potato strips. Over time, considerable increases were observed in the total microbial load and mold and yeast counts of the samples. However, the intensity of these increases was significantly higher in the uncoated sample (control) than in coated samples (*p* < .05). Thus, on the last day of storage, the control sample had a microbial load of 4.86 log CFU/g, which was 45.47%–51.24% higher than those of samples coated with Zedo gum solutions containing essential oils. Moreover, the number of molds and yeasts in the control sample totaled 102.67 CFU/g, and that in the coated samples ranged between 27.67 and 61.33 CFU/g. In general, ZE showed higher antimicrobial activity than TE.

**TABLE 4:** Total microbial load and mold and yeast counts in freshly cut potato strips during 9 days of cold storage.

Samples	Day 0	Day 3	Day 6	Day 9
Total microbial load (log CFU/g)				
Control	2.15 ± 0.17 D,a	3.37 ± 0.16 C,a	4.01 ± 0.29 B,a	4.86 ± 0.14 A,a
ZG	2.13 ± 0.14 D,a	2.64 ± 0.08 C,b	2.98 ± 0.11 B,b	3.69 ± 0.05 A,b
ZG + ZE	1.74 ± 0.22 C,b	2.03 ± 0.12 BC,c	2.12 ± 0.14 AB,d	2.37 ± 0.17 A,d
ZG + TE	1.78 ± 0.19 C,b	2.05 ± 0.10 C,c	2.35 ± 0.06 B,c	2.65 ± 0.08 A,c
Molds and yeasts count (CFU/g)				
Control	2.33 ± 1.53 D,a	22.67 ± 1.53 C,a	72.67 ± 3.22 B,a	102.67 ± 3.22 A,a
ZG	1.33 ± 0.58 D,a	6.00 ± 1.73 C,b	29.67 ± 1.53 B,b	61.33 ± 3.51 A,b
ZG + ZE	0.00 ± 0.00 D,b	1.00 ± 0.00 C,d	6.33 ± 1.16 B,d	23.33 ± 2.08 A,d
ZG + TE	0.00 ± 0.00 D,b	3.00 ± 0.00 C,c	8.67 ± 0.58 B,c	27.67 ± 1.53 A,c

*Note*: Values represent mean (*n* = 3) ± standard deviation. Different letters mean significant differences at the 5% level of probability among samples.

Abbreviations: ZG + TE, Zedo gum‐based coating containing tarragon essential oil; ZG + ZE, Zedo gum‐based coating containing *Zataria multiflora* essential oil; ZG, Zedo gum‐based coating.

In general, Zedo gum‐based edible coatings increased the resistance of potato strips against the growth of microorganisms by reducing the intensity of respiration and improving the integrity of the samples. These coatings affected the rate of growth and reproduction of microorganisms by reducing the level of available oxygen. The addition of TE and ZE to the coating solutions also limited access to oxygen. Moreover, essential oils possess remarkable antimicrobial activity, improve the protective, antibacterial, and antifungal effects of edible coatings, and delay the growth of microorganisms. Essential oils can also destroy and penetrate the outer membrane of microorganisms and drain cellular energy through the release of ATP and, ultimately, cell death (Majdinasab et al., 2020). Other researchers reported the reduction of microbial load in meats via the application of Zedo gum‐based edible coatings containing ZE (Joukar et al., 2017; Samani et al., 2022). Wu (2019) used cactus polysaccharide‐based coatings and observed a considerable reduction in the microbial load of freshly cut potatoes during the storage period. Hashemi et al. (2017) reported reduced total microbial load and mold counts in freshly cut apricots coated with basil seed gum containing oregano essential oil.

### 3.9 Overall sensory acceptance of potato strips

**FIGURE 7:**
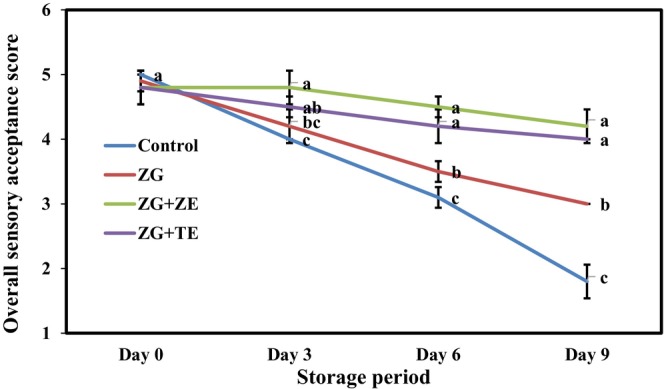
Changes in overall sensory acceptance scores of freshly cut potato strips during 9 days of cold storage. Different letters represent significant differences at the 5% level of probability among samples. ZG + TE, Zedo gum‐based coating containing tarragon essential oil; ZG + ZE, Zedo gum‐based coating containing *Zataria multiflora* essential oil; ZG, Zedo gum‐based coating.

Sensory acceptance is one of the key factors in consumers’ product selection. In this test, the overall acceptance analysis of uncoated and coated freshly cut potato strips was performed, and the related score of the samples was obtained based on appearance, odor, texture, and color (Figure 7). On the first day of cold storage, the coating of potato strips with coating solutions caused a slight decrease in the overall acceptance score. However, no statistically significant difference was observed between the control and coated samples (4.80–5.00). During the storage period, the overall acceptance score of the control sample decreased considerably and reached 1.80 at the end of the storage period. However, no substantial change was observed in the overall acceptance of the samples coated with gum solutions without essential oils until the fourth day and those coated with gum solutions containing TE and ZE until the sixth day of cold storage. These samples obtained the highest sensory acceptance score on the last day of storage (4.00–4.20). The results indicate the important effect of actively produced coatings in maintaining the sensory acceptance of freshly cut potato strips during the cold storage period.

Similarly, according to Solís‐Contreras et al. (2021), coating apple slices with guar and starch solutions containing cinnamon essential oil not only did not exhibit an adverse effect on the sensory acceptance of the samples but also maintained the sensory quality of apple slices over time. Oyom et al. (2022) also observed the improved sensory quality of peach samples coated with edible coatings containing cumin essential oil. Sarengaowa et al. (2022) showed that the use of alginate coatings containing 0.05% thyme essential oil maintained the sensory quality of freshly cut potatoes for up to 16 days.

## 4 CONCLUSION

The results of this research indicate the remarkable antioxidant and antimicrobial activities of ZE and TE. The antioxidant activity of these essential oils in both methods of DPPH radical scavenging and β‐carotene bleaching was dependent on the concentration. It showed a significant increase due to the increase in concentration. Coating freshly cut potato strips with Zedo gum‐based active coatings improved their antioxidant activity and sensory quality. It prevented weight loss, texture softening, and enzymatic browning compared with the uncoated sample during the cold storage period. These active coatings also reduced the rate of growth and reproduction of bacteria, molds, and yeasts in potato strips. In general, ZE exhibited higher antioxidant and antimicrobial activities than TE in vitro and coated potato strips. According to the results of this research, given the improved quality, microbial stability, and sensory acceptance of potato strips coated with Zedo gum‐based solutions containing ZE and TE, these active coatings can be used to extend the shelf life and maintain the quality of freshly cut potato strips. The best treatment in this research was the Zedo gum‐based solution containing ZE. Since today, due to the development of urbanization, the need to use semi‐prepared products has increased. These active coatings can be suggested to maintain the quality and extend the shelf life of freshly cut potatoes for the market.

## AUTHOR CONTRIBUTIONS

**Niyoosha Khajeh:** Formal analysis (equal); funding acquisition (equal); investigation (equal); methodology (equal); resources (equal); validation (equal); writing – original draft (equal). **Abdorreza Mohammadi Nafchi:** Conceptualization (equal); funding acquisition (equal); project administration (equal); supervision (equal); visualization (equal); writing – review and editing (equal). **Leila Nouri:** Data curation (equal); methodology (equal); project administration (equal); supervision (equal); validation (equal); visualization (equal); writing – review and editing (equal).

## CONFLICT OF INTEREST STATEMENT

The authors declare that they have no known competing financial interests or personal relationships that could have appeared to influence the work reported in this paper.

## ETHICAL APPROVAL

This study does not involve any human or animal testing.

## Data Availability

The data that support the findings of this study are available from the corresponding author upon reasonable request.
